# From Science to Success? Targeting Tyrosine Kinase 2 in Spondyloarthritis and Related Chronic Inflammatory Diseases

**DOI:** 10.3389/fgene.2021.685280

**Published:** 2021-07-05

**Authors:** Dominika Hromadová, Dirk Elewaut, Robert D. Inman, Birgit Strobl, Eric Gracey

**Affiliations:** ^1^Institute of Animal Breeding and Genetics, University of Veterinary Medicine Vienna, Vienna, Austria; ^2^Molecular Immunology and Inflammation Unit, VIB Centre for Inflammation Research, Ghent University, Ghent, Belgium; ^3^Department of Rheumatology, Ghent University Hospital, Ghent, Belgium; ^4^Schroeder Arthritis Institute, University Health Network, Toronto, ON, Canada; ^5^Departments of Medicine and Immunology, University of Toronto, Toronto, ON, Canada

**Keywords:** TYK2, spondyloarthritis, IL-23, JAK inhibitor, JAK, clinical trials

## Abstract

Spondyloarthritis (SpA) is a family of inflammatory arthritic diseases, which includes the prototypes of psoriatic arthritis and ankylosing spondylitis. SpA is commonly associated with systemic inflammatory diseases, such as psoriasis and inflammatory bowel disease. Immunological studies, murine models and the genetics of SpA all indicate a pathogenic role for the IL-23/IL-17 axis. Therapeutics targeting the IL-23/IL-17 pathway are successful at providing symptomatic relief, but may not provide complete protection against progression of arthritis. Thus there is still tremendous interest in the discovery of novel therapeutic targets for SpA. Tyrosine kinase 2 (TYK2) is a member of the Janus kinases, which mediate intracellular signaling of cytokines via signal transducer and activator of transcription (STAT) activation. TYK2 plays a crucial role in mediating IL-23 receptor signaling and STAT3 activation. A plethora of natural mutations in and around TYK2 have provided a wealth of data to associate this kinase with autoimmune/autoinflammatory diseases in humans. Induced and natural mutations in murine Tyk2 largely support human data; however, key inter-species differences exist, which means extrapolation of data from murine models to humans needs to be done with caution. Despite these reservations, novel selective TYK2 inhibitors are now proving successful in advanced clinical trials of inflammatory diseases. In this review, we will discuss TYK2 from basic biology to therapeutic targeting, with an emphasis on studies in SpA. Seminal studies uncovering the basic science of TYK2 have provided sound foundations for targeting it in SpA and related inflammatory diseases. TYK2 inhibitors may well be the next blockbuster therapeutic for SpA.

## Introduction

Spondyloarthritis (SpA) is a family of seronegative chronic inflammatory arthritic diseases, united by shared clinical features and the association with HLA-B27 (Taurog et al., 2016; Ritchlin et al., 2017). Both the axial and peripheral joints can be affected, with arthritis progression characterized by paradoxical bone erosion and new bone formation. In extreme forms, the axial joints, including the sacroiliac and intervertebral joints, can completely fuse. Inflammation of the skin, gastrointestinal tract and eyes are often present in SpA patients in the forms of psoriasis, inflammatory bowel disease (IBD) and uveitis, respectively. Intestinal inflammation, often subclinical, is common in axial forms of the disease, including the most common form, ankylosing spondylitis (AS) (Gracey et al., 2020b). Psoriasis on the other hand, is a diagnostic feature of psoriatic arthritis (PsA) (Taylor et al., 2006), which typically affects, but is not restricted to, the peripheral joints. Considerable overlap exists in the clinical phenotype of PsA and AS (Jadon et al., 2017), with modern classification criteria labeling them as peripheral SpA (pSpA) and axial SpA (axSpA), respectively (Sieper et al., 2009; Rudwaleit et al., 2011). Under these modern classification criteria, AS is a form of radiographic axSpA; however, the term AS is still in common use, so will be used throughout the manuscript where appropriate.

Therapeutic options for SpA are limited. Non-steroidal anti-inflammatory drugs (NSAIDs) remain a frontline therapy, particularly for AS (Van Der Heijde et al., 2017), with biologic and targeted synthetic disease modifying anti-rheumatic drugs (bDMARDs/tsDMARDs) acting as second line therapies when the response to NSAIDs is insufficient. For PsA, additional conventional synthetic (csDMARDs), such as methotrexate, are also recommended as frontline therapies prior to bDMARDs/tsDMARDs (Ogdie et al., 2020). Approved bDMARDS include TNF inhibitors (TNFi) and IL-17 inhibitors (IL-17i) for both AS and PsA, with IL-23 inhibitors (IL-23i) additionally approved for PsA (Al-Mossawi et al., 2019; Fragoulis and Siebert, 2020). For PsA, two types of tsDMARDs are approved: the PDE4 inhibitor, apremilast, and the Janus kinase (JAK) inhibitor, tofacitinib (Al-Mossawi et al., 2019). No tsDMARDs are currently approved for AS. With short term use, approximately 60–65% of SpA patients achieved a 20% reduction in symptoms (ASAS20) in most randomized controlled trials with current bDMARDs. With long term use, evidence exists for bDMARDs slowing joint fusion, but they may not prevent it (Haroon et al., 2013; Braun et al., 2017; Mease et al., 2018b; Koo et al., 2020).

Therapies approved for related inflammatory disease are more extensive and sometimes more effective than those approved for SpA. For rheumatoid arthritis (RA), three JAK inhibitors and a range of bDMARDs are approved, including those targeting TNFα, IL-6, IL-1β, B cells (CD20), and T cells (CD80/CD86). For IBD, the TNFi (except for etanercept) and IL-12/23i have remission rates comparable to those seen in SpA (Hanauer et al., 2002; Feagan et al., 2016). In contrast to SpA, IL-17i do not work and may worsen IBD (Hueber et al., 2012). Further, IBD has two integrin inhibitors approved, which do not appear to be effective in and may worsen SpA (Varkas et al., 2017; Dubash et al., 2018). Tofacitinib is also approved for IBD, with remission rates of around 40% (Panés et al., 2017; Sandborn et al., 2017). For psoriasis, the range of bDMARDs and tsDMARDs available is comparable to SpA, with the newer therapies, such as the IL-23i, achieving almost complete remission of skin disease in most patients (Kaushik and Lebwohl, 2019). By comparison, the same approved therapeutics for SpA generally achieve a 50% reduction in symptoms in around half of treated patients (Mease et al., 2020b).

The therapeutic armaments against SpA, and their efficacy, reflects our knowledge of the underlying immunopathology of the disease. Indeed, all approved bDMARDs and tsDMARDs for SpA were first trialed in diseases with extensively characterized immune components, such as RA or psoriasis, before being tested in SpA. In these diseases, animal models have been long established and widely used (Brand et al., 2007; van der Fits et al., 2009), and the latest omics techniques have been applied to the target tissue in humans (Zhang et al., 2019; Hughes et al., 2020). By comparison, there are few animal models of SpA that faithfully recapitulate the disease (Vieira-Sousa et al., 2015), and human target tissue, especially of the axial skeleton, remains largely inaccessible for research.

We know at the immunological level that type 3 immunity (also known as “Type-17 immunity” or the “Th17-axis”), namely IL-17A and IL-17A-producing cells, play a critical role in SpA (Taams et al., 2018; Gracey et al., 2020b). This is highlighted by the success of IL-17i across major forms of SpA. While IL-23 was long suspected to be the key driver of IL-17A in SpA, this does not seem to be the case, at least in patients with axSpA, who fail to respond to IL-23i (Baeten et al., 2018). IL-1β and IL-6 are IL-23 independent inducers of IL-17A, but biologics targeting both of these cytokines also failed clinical trials in AS (Haibel et al., 2005; Sieper et al., 2015). By comparison, IL-23 plays an essential role in all stages of psoriasis and blocking it is clinically effective (Papp et al., 2017), whereas IL-23 only seems to play a role in early experimental RA (Pfeifle et al., 2017), with its blockade providing no clinical protection against symptomatic disease in patients (Smolen et al., 2017).

HLA-B27 is a major histocompatibility complex (MHC) class I molecule that presents peptides to CD8+ T cells, allowing for their activation. By virtue of the strong association of HLA-B27 with SpA, CD8+ T cells likely play a central role in SpA pathogenesis; however, conclusive evidence from humans and animal models does not yet exist. As a result, alternative theories for how HLA-B27 may mediate a CD8+ T cell independent role in SpA have been put forward, such as the endoplasmic reticulum stress theory, and HLA-B27 interaction with killer inhibitory receptors (KIR) (Bowness, 2015). Nevertheless, a clear disturbance of cytotoxicity in CD8+ T cells of AS patients was recently revealed (Gracey et al., 2020c), and IL-17A+ CD8+ T cells expressing *IL23R* were found to be enriched in the synovial fluid of PsA patients (Steel et al., 2020). A better understanding of CD8+ T cells in SpA may facilitate the development of novel therapeutics against the diseases.

Genetic studies of SpA support the role of type 3 immunity and CD8+ T cells. Genome-wide association studies (GWAS) of AS implicate a number of genes involved in the production of, and response to, IL-17, and also many genes in MHC class I peptide processing and CD8+ T cell function (Cortes et al., 2013). PsA GWAS have not advanced as quickly as the AS GWAS, likely due to the strong overlap with psoriasis making it difficult to determine risk factors for PsA alone *vs* those shared with psoriasis. Nonetheless, a similar pattern of type 3 immunity and CD8+ T cell-related genes are linked to PsA (Bowes et al., 2015; Stuart et al., 2015). Importantly, various single-nucleotide polymorphisms (SNPs) in and around tyrosine kinase 2 (*TYK2*) were linked to both AS and PsA in these studies (Cortes et al., 2013; Bowes et al., 2015; Stuart et al., 2015; Ellinghaus et al., 2016). As TYK2, a member of the JAK family, is known to mediate intracellular signaling for the IL-23R, it is assumed that *TYK2* is one of the many genes leading to a perturbed type 3 immune response in SpA patients.

**FIGURE 1:**
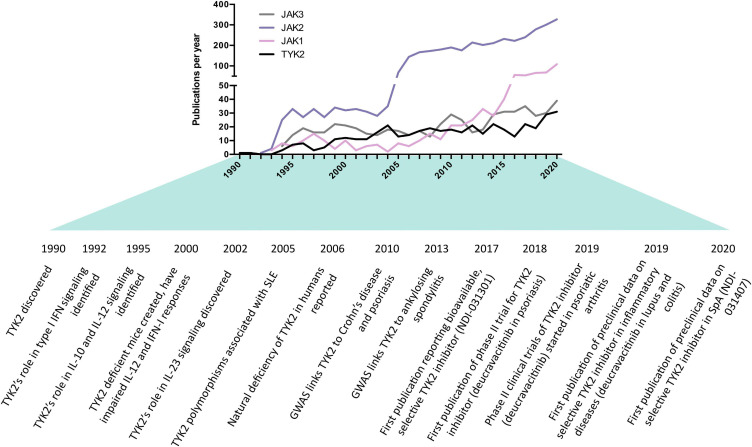
History and milestones of Janus kinase (JAK) research, with an emphasis on tyrosine kinase 2 (TYK2) in the context of SpA. Data obtained from SCOPUS database using searching the abstract and title for short name (e.g., “TYK2”) or long name (e.g., “tyrosine kinase 2”). The increase in JAK2 publications was likely driven by the discovery of JAK2 mutations in myeloproliferative disorders in 2005. The increase in JAK1 publications coincided with the discovery of its role in leukemia.

Here, we discuss the biology of TYK2 and advances made in targeting it. While this article is included in a special publication on the genetics of SpA, only a handful of genetic studies have implicated *TYK2* in the context of SpA (Cortes et al., 2013; Dendrou et al., 2016; Ellinghaus et al., 2016), and only one functional study of TYK2 has focused on SpA patients and animal models (Gracey et al., 2020a). In addition, TYK2 inhibitors are still relatively early in their development compared to other JAK inhibitors (JAKi), which is reflected in the lower number of publications on TYK2 compared to the other JAK family members (Figure 1). For these reasons, this article will more broadly discuss the biology and therapeutic targeting of TYK2 relevant to SpA, with direct citations of SpA research made when available.

## Jaks Are Essential Intracellular Cytokine Signaling Molecules

**FIGURE 2:**
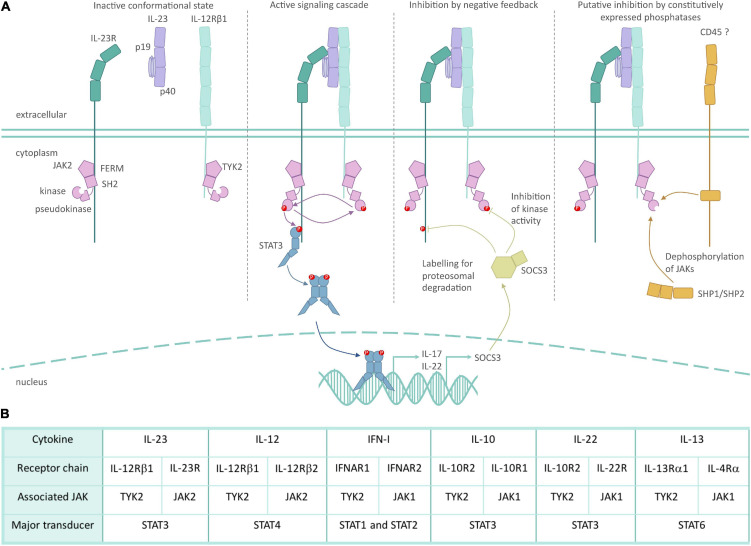
Role of TYK2 in IL-23 receptor signaling. **(A)** Inactive JAK2/TYK2 associate with the intracellular domain of their respective cytokine receptor subunits in the steady-state through FERM and SH2 domains. JAK inactivity is mediated in part by pseudokinase domain inhibition of the kinase domain. The binding of IL-23 unites its respective cytokine receptor subunits, which in turn causes a conformational change in JAK2/TYK2, allowing for their kinase activity. Phosphorylation of JAKs and the intracellular domain of the cytokine receptors allows for the recruitment and activation of the STAT3 transcription factor and subsequent induction of gene expression. Inducible inhibition of IL-23 is mediated in part by the SOCS family of proteins, which can bind to JAKs to block their cytokine activity, and can tag JAKs for ubiquitin mediated degradation. While not specifically researched in the context of IL-23R, constitutive inhibition of JAKs is mediated by phosphatases such as SHP1 and CD45. **(B)** Selected TYK2-associated cytokines, their receptor subunits and associated intracellular signaling molecules. The dominant STAT activated by the respective cytokine is indicated, but STAT usage can be cell- and context-dependent.

The JAK family of non-receptor kinases is a small but influential set of intracellular signaling molecules. Here, we illustrate key concepts of JAK mediated signaling using the IL-23R as an example to keep with the theme of this article (Figure 2). The JAK family shares the four functional domains: four-point-one, ezrin, radixin, moesin (FERM), and Src Homology 2 (SH2) domains, followed by pseudokinase and kinase domains. The peptide sequence of the FERM and SH2 domains dictate which specific cytokine receptors each JAK can bind (Haan et al., 2006; Ferrao and Lupardus, 2017). JAKs bind to their respective cytokine receptors constitutively, with JAK binding often being essential for surface expression of its cognate receptor chain (Ragimbeau et al., 2003; Kumar et al., 2008; Carbone and Fuchs, 2014). The pseudokinase and kinase domains are closely associated, with the former inhibiting the kinase activity in the resting state (Lupardus et al., 2014). The intimate association of these two kinase domains led to them being named after the Roman god, Janus, who has two faces looking in opposite directions (Wilks, 2008). The binding of a ligand to its corresponding JAK-associated receptor complex leads to conformational changes, which results in the activation of the associated JAKs through *trans*- and auto-phosphorylation, and subsequent phosphorylation of tyrosine residues of the signal transducing cytokine receptor chain (Villarino et al., 2017; Morris et al., 2018). These phospho-tyrosines act as docking sites for STAT transcription factors, which subsequently become phosphorylated by the JAKs, and translocate to the nucleus as homo- or heterodimers. Thus, JAKs are a critical link in the chain of events from cytokines to cellular responses.

There are four members of the family, namely JAK1, JAK2, JAK3, and TYK2, which mediate selected hormone, colony stimulating factor and cytokine signaling (O’Shea et al., 2015). These JAK family members partner predictably with specific cytokine receptor subunits, resulting in a consistent pairing of homo- or heterodimer JAK-receptor subunits (Morris et al., 2018). For example, the IL-12 family utilizes TYK2 through its interaction with the common receptor subunit IL-12RB1. JAK2 binds to the partner receptors, IL-23R and IL-12RB2, to provide specificity to IL-23 and IL-12, respectively. TYK2 also associates with the type I interferon receptor 1 (IFNAR1) to mediate IFN-I signaling; IL-10R2 to mediate IL-10 family signaling (IL-10, IL-22); IL-13RA1 to mediate IL-13 and in some circumstances IL-4 signaling; and gp130, the common chain of the IL-6 receptor. Despite binding to gp130, TYK2 is not required for mediating IL-6R family signaling (Wöss et al., 2019).

While JAK-STAT signaling is often portrayed as a linear path, there are multiple levels of complexity and control. JAK signaling is negatively regulated in a number of ways. Constitutively expressed phosphatases act to restrain the duration of JAK signaling, such as CD45 and SHP1/SHP2 (Morris et al., 2018), but no conclusive evidence exists if this negative regulation plays a role in IL-23R signaling. Inducible negative regulation occurs through the suppressor of cytokine signaling (SOCS) family, which can specifically block kinase activity of JAK (Liau et al., 2018), block STAT docking (Yamamoto et al., 2003) and can also target the activated cytokine receptor for degradation via ubiquitination (Kamizono et al., 2001; Durham et al., 2019). Some evidence exists that SOCS1 is able to negatively regulate IFN-I signaling via TYK2 (Piganis et al., 2011), and SOCS3 may be an important negative regulator of IL-23R signaling (Chen et al., 2006).

The STAT specificity of a given cytokine receptor is driven not by the JAKs themselves, but by the peptide sequence of the cytokine receptor’s intracellular domain providing a docking site for a given STAT (Stahl et al., 1995; Naeger et al., 1999). For example the IL-12RB2 binds STAT4, while IL-23R binds STAT3 (Teng et al., 2015; Floss et al., 2020). While a given cytokine receptor preferentially induces the activation of selected STATs, there is no clear understanding of what role the specific receptor-associated JAKs play in activating a given STAT. Our poor understanding of how these intracellular signaling pathways work is also reflected by the fact that not all phosphorylations of STATs are activating, a fact that can be overlooked in research. As an example, while STAT3 tyrosine (Y)705 is generally considered to be an indication of activation, serine (S)727 and Y640 phosphorylation negatively regulate STAT3 activity (Chung et al., 1997; Mori et al., 2017). The STAT molecules also undergo other post-translational modifications that alter their function. For example, STAT3 undergoes non-degradative ubiquitination that promotes Y705 phosphorylation (Cho et al., 2019). STAT3 also undergoes palmitoylation to promote its recruitment to the cell membrane and de-palmitoylation to release STAT3 for nuclear translocation (Zhang et al., 2020).

An additional aspect of the JAK family of kinases relevant to inhibition by small molecules is that they may also undertake kinase-independent functions. TYK2 is described to have a scaffold function in cytokine signaling receptor complexes, whereby JAK binding to a cytokine receptor chain can be essential for its surface expression (Ragimbeau et al., 2003; Kumar et al., 2008; Carbone and Fuchs, 2014). Specifically, kinase inactive (mutated) TYK2 stabilizes the receptor subunit it binds, such as IL-10R2, IL-12RB1, and IFNAR1, facilitating sustained surface expression and cytokine binding. The kinase active JAK on the partner cytokine receptor subunit can then initiate the signaling cascade (Li et al., 2013; Kreins et al., 2015). Finally, TYK2 might also act on non-STAT substrates; in the context of IFN-I signaling, phosphoinositide 3 kinase appears to be activated by TYK2 independent of its kinase activity (Rani et al., 1999). Thus, while the textbook depiction of JAK-STAT signaling often portrays a linear pathway, the truth is considerably more complicated, as is often the case in biology.

## Tyrosine Kinase 2 Deficiency and Polymorphisms in Man

**FIGURE 3:**
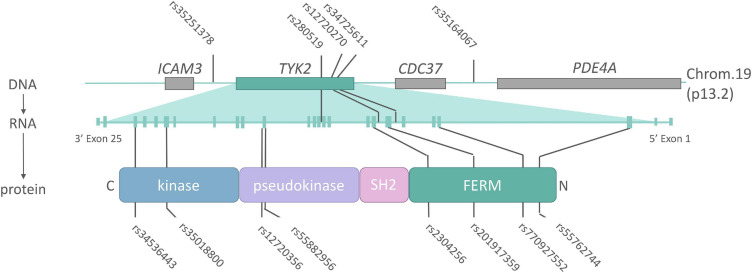
The *TYK2* locus (DNA), transcript (mRNA) and protein annotated with single-nucleotide polymorphisms (SNPs) associated with AS or related inflammatory diseases. Non-coding SNPs (intronic or intergenic) lie on top of the *TYK2* DNA locus, while non-synonymous (protein changing) SNPs indicated below TYK2 protein. Details on the indicated SNPs can be found in Table 1.

A plethora of functional mutations have been identified at the *TYK2* locus in man, ranging from complete inactivation, to mild modification of function (Figure 3 and Table 1). These “experiments of nature” have revealed the roles that TYK2 plays in specific cytokine signaling cascades, guided the development of targeted mutations in mice, and ultimately have provided the rationale for therapeutic targeting for inflammatory diseases. While most TYK2 biology is conserved between mouse and man, some important differences do exist, which will be highlighted in the next section.

**TABLE 1:** Tyrosine kinase 2 (*TYK2*) locus single-nucleotide polymorphisms (SNPs) associated with ankylosing spondylitis (AS) or related autoimmune diseases.

SNP	AA change	Location	Functional effect	MAF European	MAF Asian	OR (AS)	Association	References
rs35251378	–	Intergenic		0.2840	0.5090	N/A	PsA	Stuart et al. (2015)
rs34536443	P1104A	Exon23 kinase	Loss of kinase activity	0.0420	0.0003	0.70	Ps, RA, SLE, T1D, AS, CD, UC, and MS	Dendrou et al. (2016)
rs35018800	A928V	Exon20 kinase	Loss of kinase activity	0.0075	0.0000	0.60	RA, SLE, AS, CD, and UC	Diogo et al. (2015)
rs12720356	I684S	Exon15 pseudokinase	Loss of kinase activity	0.0863	0.0000	1.09	Ps, RA, SLE, T1D, AS, CD, and UC	Dendrou et al. (2016)
rs280519	–	Intron 11		0.5032	0.5740	N/A	Ps and SLE	Strange et al. (2010) and Cunninghame Graham et al. (2011)
rs55882956	R703W	Exon15 pseudokinase	Loss of kinase activity	0.0011	0.0280	N/A	RA (suggestive association)	Motegi et al. (2019)
rs2304256	V362F	Exon8 FERM	Promotion of exon 8 inclusion	0.2836	0.4430	N/A	Ps, SLE, and T1D	Wallace et al. (2010), Diogo et al. (2015), Enerbäck et al. (2018), and Li et al. (2020)
rs12720270	–	Intron 7	Promotion of exon 8 inclusion	0.1832	0.4700	N/A	SLE	Graham et al. (2007), Hellquist et al. (2009), and Li et al. (2020)
rs201917359	R231W	Exon7 FERM		0.0010*	0.0030*	N/A	RA	Motegi et al. (2019)
rs34725611	–	Intron 6		0.2768	0.5060	N/A	PsA	Bowes et al. (2015)
rs770927552	GCTT deletion	Exon4 FERM	Truncation (nonsense mutation)	0.0000	0.0000	N/A		Minegishi et al. (2006) and Kreins et al. (2015)
rs55762744	A53T	Exon3 FERM	Impact on protein function	0.0109	0.0006	N/A	MS	Dyment et al. (2012)
rs35164067	–	Intergenic		0.2001	0.4690	1.14	T1D and AS	Cortes et al. (2013)

Functional effect, OR (for AS only) and disease associated found in indicated reference. Minor allele frequency (MAF) obtained from ALFA project data of over 100,000 individuals on NCBI’s SNP database. *ALFA project SNP data not available, so 1,000 genomes used instead.

Tyrosine kinase 2 mutations that cause deficiency are rare, but impart a strong immune phenotype on carriers through the introduction of a premature stop codon in the *TYK2* transcript (Minegishi et al., 2006; Kreins et al., 2015; Fuchs et al., 2016). It is estimated that homozygous complete inactivation of TYK2 occurs in less than 1 in 600,000 people (Boisson-Dupuis et al., 2018). The first report of a TYK2 deficient person arose from an investigation into the cause of a patient with hyper IgE syndrome (HIES) and repeated fungal, viral and mycobacterial infections (Minegishi et al., 2006). A molecular investigation found the patient to have signaling deficiencies for IFN-I, IL-6, IL-10, IL-12, and IL-23. In particular, the patient’s T cells displayed no STAT phosphorylation or gene induction in response to IFN-I, and were incapable of making IFNy in response to IL-12. This later point was proposed as the reason why the patient had elevated Th2 and reduced Th1 cell frequency. It is important to note that IL-23-induced IL-17 in CD4+ T cells had only recently been discovered at the time of this publication in 2006 (Cua et al., 2003; Park et al., 2005), which may have been why Th17 cells were not explored. A series of case reports, published in 2015, detailed seven additional TYK2-deficient patients from five families (Kreins et al., 2015). These patients all presented with recurrent mycobacterial and viral infections, without recurrent fungal infections or HIES. As with the index TYK2 deficient patient, these subsequent individuals all had impaired responses to IFN-I, IL-10, IL-12, and IL-23, but unlike the index case, they responded normally to IL-6. Moreover, this study showed that impaired IL-6 signaling in the first patient is not due to the TYK2 deficiency. Of importance to SpA, this paper showed that despite a loss of response to IL-23, circulating Th17 cell frequencies appeared normal in TYK2 deficient individuals. A final case report, published in 2016, confirmed the essential role of TYK2 in IFN-I responses and a limited role in IL-10 activity through reduced IL-10R2 expression (Fuchs et al., 2016). The effect of this mutation on IL-12 and IL-23 signaling was not assessed.

Mutations that cause, or associate with, a reduction in TYK2 function are relatively common and can be found in and around the *TYK2* gene. Small genetic studies that focused on the *TYK2* locus itself were the first to link variants to autoimmune disease. Specifically, IFN-I related genes were targeted in studies of systemic lupus erythematosus (SLE), a rheumatic disease strongly linked to altered IFN-I activity (Sigurdsson et al., 2005). Here, the SNP rs2304256 (V362F) was found to be strongly associated with SLE (OR 1.6, corrected *P*-value 3.4 × 10^–7^). Large, unbiased genome wide association studies (GWAS) later found *TYK2* to be associated to a range of autoimmune diseases, including rs12720356 (I684S) with Crohn’s disease [odds ratio (OR) 1.12] (Franke et al., 2010) and psoriasis (OR 1.4) (Bowes et al., 2015), while AS was associated with an intergenic SNP near *TYK2* (rs35164067, OR 1.16) (Cortes et al., 2013). A large meta-analysis utilizing the GWAS data from five autoimmune diseases, including AS, confirmed the link with rs12720356 (I684S), but highlighted that the direction of association (i.e., risk *vs* protection) was not the same for each disease (Ellinghaus et al., 2016). This paradox was further explored in a meta-analysis specifically for *TYK2* by Dendrou et al. (2016), in which rs12720356 (I684S) was found to be a risk factor for AS, CD, and ulcerative colitis (UC), but not other autoimmune diseases such as RA, psoriasis, SLE and type I diabetes. The biological reason for this clustering is not clear, but may reflect the relative contribution of the kinase *vs* pseudokinase domains in the target tissues of the respective diseases. Dendrou et al. (2016) further commented that rs12720356 may not be a causative SNP in all association studies, with linked SNPs potentially providing disease risk by modulating the expression of adjacent genes. Nonetheless, the broad pattern of disease clustering based on rs12720356 resembles that seen previously in a GWAS meta-analysis of all risk factors, whereby AS was genetically closer to CD, UC, and psoriasis than to RA, SLE or diabetes (Farh et al., 2015). In addition, the aforementioned meta-analysis of *TYK2* showed the *TYK2* variant with the strongest link to all tested autoimmune diseases was rs34536443 (P1104A), whereby homozygosity imparted an OR in AS of 0.1. Below, we will give examples of how these and other natural mutations across different TYK2 domains can affect its function.

One common non-synonymous mutation in *TYK2* occurs in the pseudokinase domain, namely I684S (rs12720356). It has been reported that I684S reduces IL-12 signaling (Enerbäck et al., 2018) and that IFNy production in CD4+ T cells and NK cells, but not IL-17 production, correlates with I684S genotype (Gracey et al., 2020a). It should be mentioned that the results as to the biological effects of I684S are conflicting, with some reports disputing an effect in cell-based assays (Dendrou et al., 2016; Boisson-Dupuis et al., 2018). It is noteworthy to mention here that rs12720356 (I684S) heterozygosity was found to correlate with reduced spinal fusion in AS patients (Gracey et al., 2020a).

One of the most frequent and well-studied TYK2 mutations, P1104A (rs34536443), can be found in 4% of Europeans (NCBI dbSNP), with one in 600 Europeans being homozygous (Boisson-Dupuis et al., 2018). This missense mutation in the catalytic domain causes a loss of TYK2 kinase activity, yet the protein is fully translated (Li et al., 2013). Akin to the TYK2 deficiency discussed above, individuals homozygous for P1104A are susceptible to mycobacterial infections and do have impaired IL-23 signaling (Dendrou et al., 2016; Boisson-Dupuis et al., 2018; Kerner et al., 2019). Data on the functional effects of P1104A beyond IL-23 are conflicting: One paper reported P1104A transduced EBV T and B cells and primary human cells have only a modest interference with IFN-I and IL-10 signaling and no alteration in IL-12 signaling (Boisson-Dupuis et al., 2018). Another study of P1104A reported an effect on IFN-I and IL-12 induced STAT phosphorylation, but no effects on IL-10 or IL-6 signaling in primary human cells (Dendrou et al., 2016). Both studies reported no impact of P1104A on the surface receptor expression of IFNAR1, IL-12R, and IL-23R.

The IL-23 specific effect of TYK2 was recently replicated in a study of two siblings with compound heterozygous mutations in the TYK2 FERM domain (Nemoto et al., 2018). These patients were heterozygous for both the index truncation mutation discussed above (Minegishi et al., 2006) (now designated rs770927552), and a novel non-synonymous mutation (rs201917359, R231W). It was estimated that the protein levels of TYK2 were reduced by 35% in these subjects; however, IFN-I, IL-6, IL-10, and IL-12 signaling remained intact. It is likely that R231W results in altered binding of TYK2 to the cytokine receptors given its location in the FERM domain. Indeed a recent study demonstrated reduced IL-23 signaling activity in a cell based assay for R231W (Motegi et al., 2019), yet in neither study was surface cytokine receptor expression assessed.

Finally, it is possible that common variants in the *TYK2* locus can also affect *TYK2* splicing. One recent study addresses this concept, in which two SNPs were found to cause alternative splicing of TYK2 (Li et al., 2020). Specifically, the intronic rs12720270 and exonic rs2304256 (V362F) were found to promote the inclusion of exon 8 in the *TYK2* transcript. The same study showed exon 8 not to affect kinase activity of TYK2, but to be essential for cell responsiveness to IFN-I. These results can be best explained by location of exon 8 in the FERM domain, thus these splice variants impact on association of TYK2 with cytokine receptors, but not its catalytic activity.

In summary, nonsense mutations of *TYK2* resulting in complete loss of TYK2 protein have strong immunological phenotypes by affecting the intracellular signaling of a range of cytokines both directly through kinase activity of TYK2, and indirectly through its scaffolding function. On the other hand, missense mutations which keep TYK2 protein largely intact, but selectively affect its function, appear to have a more restricted effect on cytokine signaling, with the strongest known effects being on IFN-I, IL-12, and IL-23.

## Using Mice to Study TYK2: Pros and Cons

Mice play a crucial role in biomedical research, yet approximately 96 million years of divergent evolution have resulted in biological discrepancies when using animals to understand human biology (Nei et al., 2001; Mestas and Hughes, 2004). While many biological roles of TYK2 between mouse and man are conserved, there are some crucial differences that must be considered. Here, the development of mice to study TYK2 will be discussed, before comparing the biological function and effects of TYK2 in mice *vs* man.

Human TYK2 and murine Tyk2 share approximately 80% sequence identity at the mRNA level and 85% at the protein levels (NCBI blast). While this may seem low, it is on par with the average conservation of orthologous genes between the species (Makałowski et al., 1996). To this point, one recent study examined the inter-species sequence homology of the Tyk2 ATP binding site in the kinase domain, revealing only one amino acid substitution out of 42 amino acids between species (Gerstenberger et al., 2020b). Here, human isoleucine (I) 960 was found to be substituted by a valine in mouse at the equivalent position (V980). While the functional effect of this single amino acid change on the activity of Tyk2 *in vitro* and *in vivo* is not known, it was found to have a clear effect on the potency of selective TYK2 inhibitors as discussed in the following section.

The first manipulations of Tyk2 in mice were complete knockouts. Tyk2 KO mice were shown to have defective IFN-I and IL-12 responses, and were susceptible to viral infection, albeit not as severely as IFNAR1 KO mice (Karaghiosoff et al., 2000; Shimoda et al., 2000). IL-10 signaling was shown to be impaired only in certain experimental conditions (Shaw et al., 2006) and no effect was seen on IL-6 signaling (Karaghiosoff et al., 2000; Shimoda et al., 2000). These initial studies preceded the discovery of IL-23, with subsequent studies using Tyk2 KO mice revealing a crucial role for this JAK in IL-23 signaling (Nakamura et al., 2008), including an essential role in models for both dermatitis and colitis (Ishizaki et al., 2011).

Following the characterization of complete Tyk2 KO mice, loss of function mutations were reported. The first was a spontaneous mutation in the B10.Q/J strain (Shaw et al., 2003). This mouse strain was noted for its lack of response to IL-12 (Yap et al., 2001), leading to genomic studies pinpointing the mutation to a SNP in the pseudokinase domain rendering Tyk2 non-functional (Shaw et al., 2003). Importantly, this pseudokinase mutation completely blocked STAT3 phosphorylation in response to IL-23. The second loss of function mutation to be reported was kinase inactivation through targeted single base pair mutation (K923E) (Prchal-Murphy et al., 2012). These mice have defective IFN-I and IL-12 response akin to complete Tyk2 KO mice (Prchal-Murphy et al., 2015). Importantly, it was observed that the Tyk2-K923E mice also had drastically reduced protein levels of Tyk2 despite transcript levels being unaffected, suggesting an important role for kinase activity of Tyk2 on its stability in mice (Prchal-Murphy et al., 2012), an effect not seen in humans. A third targeted mutation of Tyk2 was designed to mimic P1104A (rs34536443) through mutating the orthologous proline at 1124 (P1124A) (Dendrou et al., 2016). Unlike the Tyk2-K923E mice, Tyk2-P1124A mice had normal transcript and protein levels of Tyk2. Despite this, the Tyk2-P1124A mice also had defective IFN-I, IL-12, and IL-23 responses.

The take home message from these murine models of altered Tyk2 function is that, as with humans, IFN-I, IL-12 and IL-23 pathways are undeniably reliant on Tyk2. Care must be taken when selecting Tyk2 mutated mice as to which particular model to use for the question to be answered; complete knockout, kinase silencing, or loss of function through targeted mutation. For certain applications, inter-species differences may also need to be considered, such as that occurring in the ATP binding site of Tyk2.

## Tyrosine Kinase 2, A Once Neglected Therapeutic Target

The hunt for selective inhibitors for JAKs began in the mid 1990’s as a collaboration between Pfizer and the NIH (Garber, 2013). The general strategy at the time was to generate ATP-mimics that would block the kinase domain of JAKs, and thus inhibit their function. The first generation inhibitors were relatively non-specific, blocking all members of the JAK family with various potencies; tofacitinib mainly targeted JAK1/JAK3, baricitinib JAK1/JAK2, and ruxolitinib JAK1/JAK2 (Banerjee et al., 2017). The second generation of JAKi for inflammatory diseases were designed to avoid JAK2, as inhibition of this molecule was found to induce cytopenia, a serious adverse event. JAK1 was the favored target, owing to its broad involvement in signaling by inflammatory disease-relevant cytokines, including the IL-2, IL-6, IL-13, type I and type II IFN families (Schwartz et al., 2017). Two promising examples of JAK1 inhibitors approved for use in rheumatic diseases are filgotinib and upadacitinib (Choy, 2019). TYK2 has long been touted as an ideal therapeutic target for inflammatory diseases due to its association with a small number of cytokine receptors relative to the other JAKs, and central role in blocking IL-23 (Shaw et al., 2003; Ishizaki et al., 2014). Despite this, the development of selective TYK2 inhibitors lagged behind the progress made by other second generation JAKi. That is not to say that attempts at inhibiting TYK2 have not been made: the patent history for TYK2 reveals a number of approaches made by academic groups and pharmaceutical companies to target this JAK (Menet, 2014; He et al., 2019), but this activity is not reflected in the literature, with relatively few papers published for TYK2 inhibitors.

Targeting of TYK2 by small molecule initially followed the same strategy as with the other JAKs, namely targeting of the kinase domain (“catalytic inhibitors”). A notable example of such a kinase domain inhibitor is NDI-031407, which effectively blocked IL-23-induced skin inflammation and SpA-like arthritis in mice (Gracey et al., 2020a). In addition, there have been publications involving dual TYK2 and JAK1 catalytic inhibitors; SAR-20347, blocked psoriasis-like skin inflammation in mice (Works et al., 2014) and PF-06700841 (brepocitinib), was found to be protective against adjuvant-induced arthritis in rats (Fensome et al., 2018). Despite this apparent success, this line of TYK2/JAK1 dual inhibitors were found to have drastically reduced potency against murine and rat Tyk2 compared to human TYK2 due a single amino acid substitution in the ATP binding site (Gerstenberger et al., 2020b), as discussed above. Using the humanized Tyk2-V980I mouse, a TYK2-selective inhibitor, PF-06826647, was shown to be effective against murine dermatitis (Gerstenberger et al., 2020a). Thus, it is possible that the relatively slow progress in targeting catalytic domain of TYK2 was in part caused by this previously overlooked inter-species variation.

A novel approach was recently taken to target not the kinase domain, but the pseudokinase domain of TYK2. These so called “allosteric” inhibitors act by stabilizing the pseudokinase domain of TYK2, thus promoting auto-inhibition of kinase domain of TYK2 (Tokarski et al., 2015; Wrobleski et al., 2019). BMS-986165 (deucravacitinib) was the first and most advanced molecule to take this approach (Burke et al., 2019; Wrobleski et al., 2019), revealing unprecedented specificity for TYK2 over the other JAKs (>10,000×). The preclinical and clinical trials involving this molecule will be discussed in the following section. A second pair of pseudokinase inhibitors, TYK2iA and TYK2iB, have been developed with available data demonstrating strong inhibition of IL-12 and IFN-I in cellular assays in the context of type I diabetes (Coomans de Brachène et al., 2020). These allosteric inhibitors have yet to be tested for their ability to block IL-23, or inhibit the rheumatic diseases.

Taken together, TYK2 inhibition by small molecule appears to have come of age. Initial targeting of the kinase domain yielded promising lead molecules, and provided preclinical evidence that TYK2 is a valid target. However, taking the novel approach of targeting the pseudokinase domain revolutionized the field. This is evident in the success of early stage clinical trials, and the initiation of phase III trials in various inflammatory diseases that will be discussed in the next section.

## Preclinical and Clinical Trials of TYK2 Inhibitors

While there have been a number of publications detailing preclinical *in vitro* and *in vivo* experiments with a range of TYK2 inhibitors, few have progressed to clinical trials. Of those in trials, the primary indication has been psoriasis, owing to its strong link to IL-23, and unambiguous primary clinical endpoint, namely resolution of skin inflammation. Here we will discuss the preclinical and clinical data pertaining to TYK2 inhibitors that have focused on the type 3 immunity in SpA and related inflammatory diseases. For completeness, we will also discuss first generation JAKi that block TYK2 as a secondary target, and second generation non-TYK2 targeting JAKi that have completed advanced clinical trials in SpA (Table 2).

**TABLE 2:** Janus kinase (JAK) inhibitors and their specificity for TYK2.

Name	Other name	Primary JAKs	Selectivity (cell free kinase assay)		References
Tofacitinib	CP-690550	JAK3/JAK2	21× (JAK3 *vs* TYK2)		Meyer et al. (2010)
Baricitinib	INCB-28050/LY-3009104	JAK2/JAK1	10× (JAK2 *vs* TYK2)		Fridman et al. (2010)
Upadacitinib	ABT-494	JAK1	100× (JAK1 *vs* TYK2)		Parmentier et al. (2018)
Filgotinib	GLPG0634	JAK1	12× (JAK1 *vs* TYK2)		Van Rompaey et al. (2013)
Brepocitinib	PF-06700841	JAK1/TYK2	3.3× (TYK2 *vs* JAK2)	282× (TYK2 *vs* JAK3)	Fensome et al. (2018)
Deucravacitinib	BMS-986165	TYK2	5× (TYK2 JH2 *vs* JAK1 JH2)	>10,000× (all JAK JH1 domains, JAK2, and JAK3 JH2)	Wrobleski et al. (2019)

*For non-TYK2 selective JAKi, selectivity of primary targeted JAK *vs* TYK2 given. For TYK2-selective inhibitors, selectivity of TYK2 *vs* off target inhibition of other JAKs given. Note that for first generation JAKs (tofacitinib and baricitinib), secondary JAKs are effectively blocked at working JAK concentrations, and thus these JAKi are often referred to as “pan-JAK” inhibitors.*

As early as 2015, the first TYK2-selective inhibitors were displaying exciting preclinical results. Conference abstracts on the catalytic inhibitors, NDI-031407 and its predecessor NDI-031301, revealed potency for IL-12 signaling blockade *in vitro*, and IL-23-induced dermatitis *in vivo* (Miao et al., 2015, 2016). NDI-031407 was recently shown to block both human and murine IL-23R signaling and IL-17 induction *in vitro*, and was successful in limiting Th17 cell expansion and halting the development of experimental murine SpA *in vivo* (Gracey et al., 2020a). Importantly, despite TYK2 being essential for IL-23-induced STAT3 phosphorylation and IL-22 production, TYK2 was only partially responsible for IL-23-induced IL-17. Successors to these molecules are now in phase I clinical trials (Bristol Myers Squibb, 2021).

Pfizer, who developed one of the most widely used JAKi to date, tofacitinib, has been active in developing TYK2 catalytic inhibitors. As early as 2014, phase I clinical trials (NCT02310750) had begun on the TYK2/JAK1 dual inhibitor brepocitinib (PF-06700841), although preclinical or clinical data was not published until 2018 (Banfield et al., 2018; Fensome et al., 2018). Preclinical data on brepocitinib showed it to be effective at blocking IL-23 signaling in human *in vitro* cellular assays, and blocked adjuvant-induced arthritis in rats (Fensome et al., 2018). Brepocitinib has since done well in both phase I and phase II trials, whereby it reduced C-reactive protein (CRP) by almost 50% and almost completely resolved skin inflammation in psoriasis patients (75% reduction in their psoriasis symptoms [PASI75] in ∼80% of subjects) (Banfield et al., 2018; Forman et al., 2020). Of importance to SpA, brepocitinib is currently in phase II trials for PsA (NCT03963401). In addition, Pfizer has also developed a TYK2-specific catalytic inhibitor, PF-06826647. This molecule has been shown to block IL-23 *in vitro*, and can suppress imiquimod-induced dermatitis in Tyk2-V980I mice (Gerstenberger et al., 2020a). While phase I trials only started in 2017 (NCT03210961), they revealed a reasonable safety profile, and a clinical effect against psoriasis (Tehlirian et al., 2020). Phase II trials are ongoing for PF-06826647 in various inflammatory diseases, yet no forms of SpA are currently being investigated as targets.

The most advanced TYK2 inhibitor is deucravacitinib (BMS-986165). As previously discussed, this inhibitor targets the pseudokinase domain. As early as 2016, conference abstracts of preclinical data on deucravacitinib revealed it to be effective at blocking IL-23 signaling, and showed that it could prevent experimental colitis in mice (Gillooly et al., 2016). These preclinical results were recently published, demonstrating the ability of deucravacitinib to block IL-23-induced STAT3 phosphorylation in human CD4+ T cells and IL-17 production in murine CD4+ T cells (Burke et al., 2019). The same publication showed that this TYK2 inhibitor could prevent colitis in two distinct murine models and a partner publication demonstrated the ability of deucravacitinib to prevent IL-23-induced skin inflammation in mice (Wrobleski et al., 2019). There are no publications that detail efficacy of deucravacitinib in preclinical models of SpA or rheumatoid arthritis, but it is effective in blocking IFN-I dependent lupus-like disease in mice (Burke et al., 2019). Phase I trials with deucravacitinib began in 2017, with phase II trials cumulating in astounding results for the treatment of psoriasis (Papp et al., 2018). Specifically, 75% of patients achieved a PASI75 and almost 50% achieved a 90% reduction in skin scores (PASI90). Of considerable importance to SpA, the results of a phase II trial of 200 PsA patients treated with deucravacitinib were recently presented, in which 63% of patients achieved a 20% improvement in rheumatic symptoms (ACR20), compared to 32% in the placebo group (NCT03881059) (Mease et al., 2020).

While not yet compared head-to-head against biologics or other JAKi, deucravacitinib looks to be a solid candidate for treatment of inflammatory diseases. Specifically, deucravacitinib achieved primary endpoints comparable to the TNFi trials for psoriasis, yet is less effective than anti-IL23 blockers (Reich et al., 2019), which achieve PASI90 in 90% of patients. In addition, deucravacitinib appears to be more effective against psoriasis than existing JAK inhibitors such as tofacitinib and baricitinib, likely due to more specific targeting of IL-23 (Papp et al., 2016a,b). Importantly, this phase II trial of deucravacitinib did not report any of the common side effects of current JAKi, such as cytopenia, likely due to avoidance of JAK2 inhibition.

There are currently no clinical trials reported for TYK2 inhibition in axSpA. Given IL-23i failed to demonstrate a meaningful effect against AS (radiographic axSpA) (Deodhar et al., 2019) and broad axSpA (Baeten et al., 2018), it is not clear if blocking this pathway by TYK2 will be beneficial for this group of diseases. The failure of IL-23i for axSpA has created an enigma in the field as anti-IL-23 is effective against pSpA (PsA) (Mease et al., 2020b), and related skin (Reich et al., 2019) and gut diseases (Feagan et al., 2016, 2017). While there are many possible explanations for this discrepancy (Siebert et al., 2018; McGonagle et al., 2021), there has been no conclusive evidence at the molecular level as to why IL-23i failed in axSpA. The use of TYK2 inhibitors against axSpA to target the IL-23 axis from a different angle, may shed light on this currently unexplainable conundrum.

One concern with blocking TYK2 is the occurrence of viral and mycobacterial infections as predicted by individuals with TYK2 deficiency (Kreins et al., 2015; Kerner et al., 2019). While mycobacteria infections were not reported in the trials of deucravacitinib, there was a higher incidence of respiratory and nasopharyngeal infections, indicating enhanced susceptibility to viral infection as an adverse event to TYK2 blockade. Of note, an expert committee was recently formed to evaluate existing data on JAKi in order to provide guidance for the use of JAKi in the treatment of the rheumatic diseases (Nash et al., 2020). This report concluded that given the available safety profiles, serious infections do occur with JAKi at a rate comparable to the TNF inhibitors. Only tofacitinib presents an enhanced risk for infection relative to the TNFi in patients over 65 (Lortholary et al., 2020; Nash et al., 2020).

For completeness, it is important to mention that non-TYK2 targeting JAKi have been generating considerable interest in SpA, with many achieving encouraging results in clinical trials. Proof of JAKi effectiveness in axSpA came from an off-label, proof of concept phase II trial of tofacitinib in AS (van der Heijde et al., 2017). This pan-JAK inhibitor achieved 20% improvement in AS symptoms (ASAS20) in 80% of patients at the higher dose (*vs* 40% in placebo), and was able to reduce MRI scores of the sacroiliac joint. Based on the success of this trial, tofacitinib entered phase III trials, which were recently completed (NCT03502616). Conference proceedings of these advanced trials appear to have confirmed the successes of the phase II trial, with 56% achieving ASAS20 *vs* 29% in the placebo group, and 40% achieving a 40% improvement in symptoms (ASAS40) *vs* 12.5% in the placebo (Deodhar et al., 2020). These levels are slightly lower than phase III trials with IL-17i, which report an ASAS20 of 70% (Marzo-Ortega et al., 2017). PsA was also proven to be effectively treated with tofacitinib, achieving an ACR20 of 50% *vs* 24% in the placebo group (Gladman et al., 2017). On the basis of such results, tofacitinib is approved for use in PsA, with applications submitted to regulatory agencies for use in AS.

The success of tofacitinib in SpA spurred interest in treating SpA with second generation JAKi. The JAK1 selective inhibitor, filgotinib, reached primary endpoints in phase II trials of both AS and PsA (Mease et al., 2018a; van der Heijde et al., 2018). In both forms of SpA, this JAK1 inhibitor appear on par with, if not superior to, tofacitinib, achieving an ASAS20 of 76% for AS and an ACR20 of 80% for PsA. Head-to-head trials are required to confirm this. Phase III trials recently begun for filgotinib treatment of AS (NCT04483700 and NCT04483687). Upadacitinib, also a selective JAK1 inhibitor, displays similar efficacy against SpA. In PsA, a phase III trial revealed reasonable success, with 64% of the treated group reaching an ACR20 (Mease et al., 2020a). In AS, a combined phase II/III trial demonstrated an ASAS20 of 65%, and significant improvements in both spine and sacroiliac MRI scores (van der Heijde et al., 2019). Based on these successes, approval for upadacitinib treatment of axSpA has been requested to both the FDA and EMA, with a decision expected in 2021. Without a doubt, the therapeutic armament against SpA will expand over the next 5 years with the addition of multiple JAKi.

## Conclusion

With current approved therapeutics for SpA failing to fully prevent disease progression despite providing symptomatic relief and some modulation of progression of structural damage with TNFi (Haroon et al., 2013; Koo et al., 2020; Sepriano et al., 2021), there is a clear unmet medical need for novel DMARDs. TYK2 is implicated in multiple chronic inflammatory diseases, including SpA, through genetic associations and a central role in IL-23 activation of innate, innate-like and adaptive immune cells. Further, TYK2 is an attractive therapeutic target as it mediates more selective intracellular cytokine signaling compared to other JAKs. Despite depictions of TYK2 as an on/off switch, biochemical and immunological studies in mouse and man reveal considerable complexity in the regulation of TYK2, and kinase-dependent *vs* -independent functions. This complexity is highlighted by diverse functional and immunological effects of natural mutations throughout the *TYK2* gene, with selected SNPs having opposing risk *vs* protective effects in different autoimmune diseases. While the tools exist to understand the functional complexity of TYK2 in mice, caution must be taken when selecting the correct mouse model for the question at hand, given both the biological differences between murine and human TYK2, and TYK2 kinase-dependent and -independent functions.

Janus kinase inhibitor appear set to disrupt the 15-years dominance that cytokine-blocking mAb have had on the treatment of SpA. Blocking JAK1 and all its associated cytokines appear to be as effective as the current approved biologics for SpA. Time will tell if the oral availability of such a JAK inhibitors will provide a competitive advantage against established injectable biologics. While TYK2 inhibitors are relatively early in their development as compared to other JAKi, they appear to now be coming of age. Available data suggests that TYK2 blockade will be effective at treating forms of SpA such as PsA, with a safety profile comparable, if not superior to, other JAK inhibitors. Preclinical data strongly supports a role for TYK2 in axSpA, and thus TYK2 inhibitors are poised to resolve the paradoxical failure of the IL-23i in axSpA. Time will tell if the science behind TYK2 inhibition will prove successful in SpA.

## Author Contributions

EG and DH planned and wrote the manuscript. DE, RI, and BS critically reviewed the manuscript. All authors contributed to the article and approved the submitted version.

## Conflict of Interest

The authors declare that the research was conducted in the absence of any commercial or financial relationships that could be construed as a potential conflict of interest.
